# Clinicopathologic characteristics, metastasis-free survival, and skeletal-related events in 628 patients with skeletal metastases in a tertiary orthopedic and trauma center

**DOI:** 10.1186/s12957-021-02169-7

**Published:** 2021-02-25

**Authors:** Georg Herget, Babak Saravi, Eugenia Schwarzkopf, Mara Wigand, Norbert Südkamp, Hagen Schmal, Markus Uhl, Gernot Lang

**Affiliations:** 1 Department of Orthopedics and Trauma Surgery, Medical Centre–Albert-Ludwigs-University of Freiburg, Faculty of Medicine, Albert-Ludwigs-University of Freiburg, Hugstetterstrasse 55, 79106 Freiburg, Germany; 2 Department of Orthopaedic Surgery, Odense University Hospital, Sdr. Boulevard 29, 5000 Odense, Denmark; 3 Department of Radiology, Pediatric Radiology, and Interventional Radiology, St. Josefskrankenhaus, Sautierstraße 1, 79104 Freiburg, Germany

**Keywords:** Cancer, Metastases, Metastatic bone disease, Skeletal-related events, SRE, Pathological fracture, Spine, Complications, Tumor board, Follow-up

## Abstract

**Background:**

Skeletal-related events (SREs) due to bone metastases (BM) significantly impact the morbidity and mortality of cancer patients. The present study sought to investigate clinicopathological characteristics, metastasis-free survival (MFS), and SREs in patients referred to a tertiary orthopedic and trauma center.

**Methods:**

Data were retrieved from electronic health records (*n*=628). Survival curves were estimated utilizing the Kaplan–Meier method. The Cox regression model was used to determine factors influencing MFS based on estimated hazard ratios (HRs).

**Results:**

Breast (55.8%) and lung (18.2%), and lung (32.9%) and prostate (16.8%) cancer were the most common cancer types in our cohort in women and men, respectively. Fifteen percent of patients presented with BM as the first manifestation of tumor disease, 23% had metastasis diagnosis on the same day of primary tumor diagnosis or within 3 months, and 62% developed BM at least 3 months after primary tumor diagnosis. Osteolytic BM were predominant (72.3%) and most commonly affecting the spine (23%). Overall median MFS was 45 months (32 (men) vs. 53 (women) months). MFS was shortest in the lung (median 15 months, 95% CI 8.05–19) and longest in breast cancer (median 82 months, 95% CI 65.29–94). Age (≥ 60 vs. < 60 years) and primary cancer grading of ≥2 vs. 1 revealed prognostic relevance.

**Conclusion:**

Women with breast or lung cancer, men with lung or prostate cancer, age ≥60 years, male sex, and primary cancer grading ≥2 are associated with increased risk for MBD. Intensified follow-up programs may reduce the risk of SREs and associated morbidity and mortality.

## Background

Approximately 1.2 million patients per year are diagnosed with cancer in the USA [1]. Metastatic bone disease (MBD) occurs during the course of the disease in 50–70% of all tumor patients and can cause severe skeletal-related events (SRE) [2]. Breast (65–75%), prostate (65–75%), bronchial (30–40%), renal cell (20–25%), and thyroid carcinomas (60%) often metastasize into the skeletal system along with other visceral metastases and are therefore relevant determinants for the therapeutic strategy [3, 4]. Furthermore, osseous metastases are frequently the first manifestation of a malignant tumor disease [5, 6].

Due to significant improvements in the diagnosis and therapy of many malignancies, the survival time of tumor patients has steadily increased in the last years and will much likely advance [7, 8]. Thus, MBD can be a chronic condition, as most of the patients with bone metastases are incurable [7, 9, 10]. Bone metastases (BM) often remain asymptomatic for a long time or present with non-specific symptoms until complications such as pathological fractures, e.g., occur. Consequently, early detection, as well as an interdisciplinary stage-adjusted therapy of bone metastases, has recently gained attention to avoid complications like SREs. SREs are associated with increased morbidity, decreased function in activities of daily living (ADLs), overall decreased quality of life, and, finally, mortality [3, 11].

Profound knowledge of specific tumor characteristics and prognostic factors for predictive risk stratification models may help prevent severe SREs and reduce subsequent morbidity and mortality. Therefore, the present study sought to determine clinicopathologic characteristics, metastasis-free survival (MFS), and skeletal-related events of patients who were referred to our orthopedic oncology outpatient clinic due to osseous metastases.

## Patients and methods

### Ethical considerations

The study was approved by the local institutional review board (protocol number 224/16) at the University Medical Center Freiburg, Freiburg, Germany. All studies were performed in accordance with the ethical standards as laid down in the 1964 Declaration of Helsinki and its later amendments or comparable ethical standards.

### Study population

We conducted a single-center retrospective cohort study of 628 patients at our university medical tertiary orthopedic and trauma center and comprehensive cancer center, respectively. The main inclusion criteria were skeletal metastases as the cause of presentation. Patients with lymphoma and/or multiple myeloma were excluded. Before attending the oncologic, orthopedic outpatient clinic, patients underwent a standardized diagnostic algorithm according to current guidelines for the diagnosis and treatment of the respective tumor entity. In patients who had metastases as the first site of diagnosis, these diagnostic algorithms were performed after BM diagnosis.

### Data management

To assess clinical parameters, oncologic characteristics (histopathology, tumor grading, the time point of diagnosis of the primary tumor and associated bone metastases (BM), and localization of osseous lesions) and SREs were extracted from our local hospital information system. Patients were assigned into three groups depending on the occurrence of BM to examine differences in the frequency of clinicopathologic characteristics and SREs: (1) patients with detection of metastases before primary cancer diagnosis (“primary bone metastases,” PBM group), (2) patients with the diagnosis of metastases at the same time or within 3 months after the primary cancer diagnosis (“primary cancer with bone metastases,” PSBM group), and (3) patients with bone metastases > 3 months after the primary cancer diagnosis (“bone metastases during follow-up,” FBM group). Furthermore, BM were stratified by localization as well as osteolytic, osteoblastic, or mixed osteolytic-osteoblastic characteristics, respectively. The localization of osseous lesions was separated into six groups for statistical analysis: spine, spine and pelvis and others, spine and others, pelvis, pelvis and others, others (upper extremity, lower extremity, skull, rib, sternum, clavicle, scapula, multiple localizations, or combinations of the aforementioned localizations). SREs were divided into the following four groups for statistical analysis: (1) fracture with neurological complications, (2) fracture without neurological complications, (3) neurological complications without fracture, (4) no complications. The majority of malignant primary tumors were graded according to the Union International Contre le Cancer (UICC) classification [12]. For histologic grading of prostate cancer, Gleason Score was used [13]. A Gleason score ≤ 6 was defined as low-grade cancer, a Gleason score of 7 as medium-grade cancer, and a Gleason score ≥ 8 was defined as high-grade cancer [14]. Three groups were established to examine the prognostic relevance of primary cancer grading scores with regards to MFS: tumor grading score 1 (patients with a UICC grade 1 or a Gleason score of ≤ 6), tumor grading score 2 (patients with a UICC grade 2 or a Gleason score of 7), and tumor grading score 3 (patients with a UICC grade of 3 or a Gleason score of ≥ 8). Patients with melanoma were excluded from this examination as the grading data did not allow an assignment to either of these groups. Treatment data were extracted to analyze the distribution of the following therapies for descriptive statistics: conservative treatment (e.g., orthotic braces or prolonged bed rest), surgery, radiotherapy (RTx), chemotherapy (CTx), and the combination of the aforementioned. Subsequently, three groups were built: patients who underwent surgery, local radiotherapy, and chemotherapy, respectively; patients who underwent only surgery, or patients who did not undergo any surgery (nonsurgical group).

### Statistical analysis

Descriptive statistics was performed to assess and compare distributions of clinicopathological factors. Median is shown with its 95% confidence interval (95% CI). To describe the boxplots, the median is shown with its interquartile range (IQR). Kaplan-Meier curves and log-rank tests were used for metastasis-free survival analysis in the FBM group. Multivariate analysis was performed to investigate the relationship between MFS and sex, age, the grade of the primary tumor, and the primary cancer types in the FBM population. A non-proportionality test based on Schoenfeld residuals was conducted to check for proportional hazards assumption. If the assumption was fulfilled, the Cox proportional hazard regression model was used to calculate the hazard ratios (HRs) and its 95% confidence interval. A two-sided *p*-value < 0.05 was considered to be statistically significant. Statistical analysis was performed using Stata Statistical Software Release 15 (StataCorp. 2011, College Station, TX, USA).

## Results

### Basic demographics and clinicopathological characteristics

**Fig. 1:**
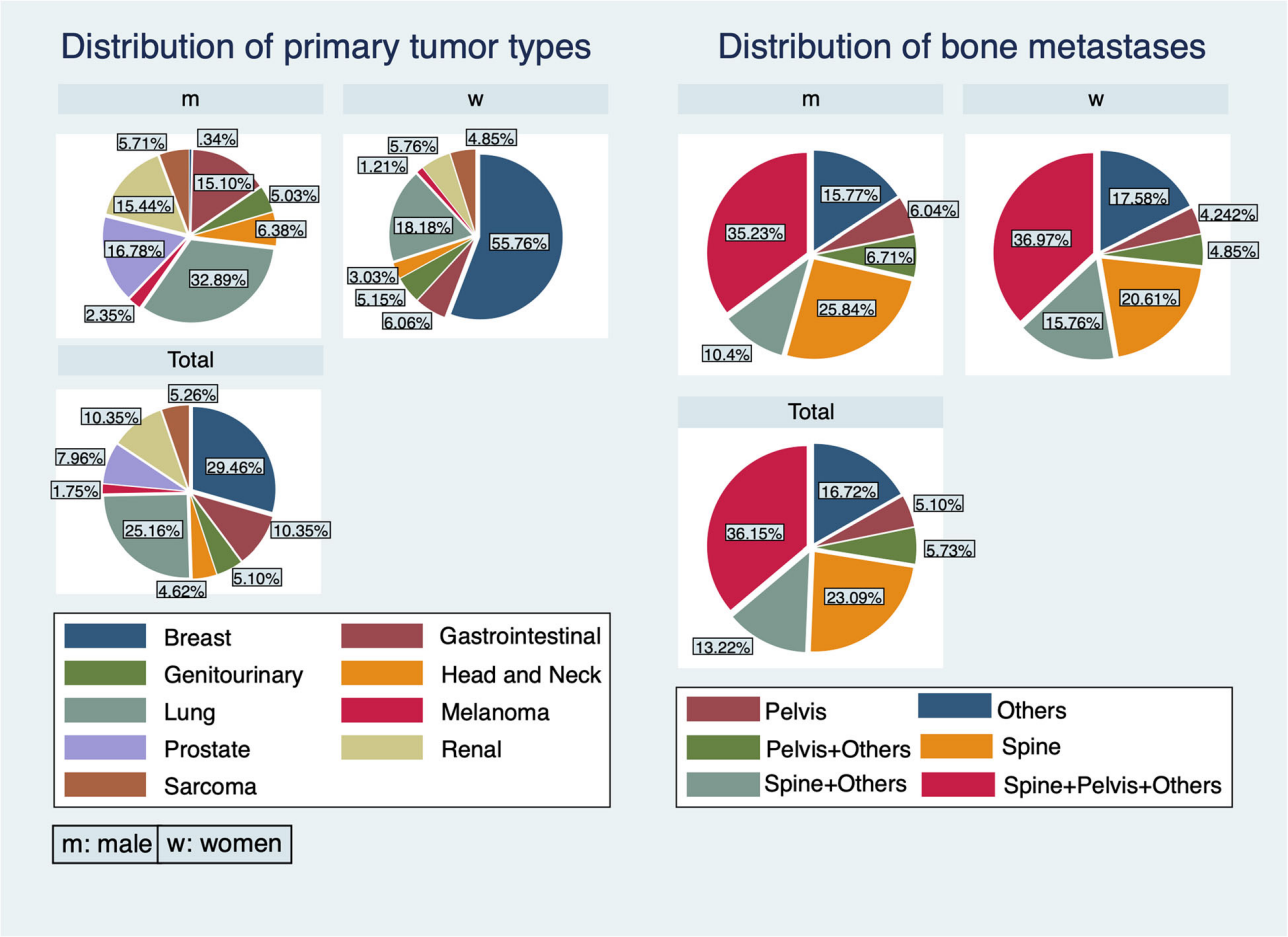
Distributions of tumor types and bone metastases (*n*=628). The most prevalent tumor type in women was breast cancer (55.76%), followed by lung cancer (18.18%). The most prevalent tumor type in men was lung cancer (32.89%), followed by prostate cancer (16.78%). Bone metastases were most often seen in multiple locations (spine+pelvis+others). Other sites than the spine and pelvis “Others” included upper extremity, lower extremity, skull, rib, sternum, clavicula, scapula, multiple localizations, or combinations of the aforementioned localizations

The present study population comprised 330 females (52.6%) and 298 males (47.5%) (Table 1). The median age for all patients was 61 years (range 2–92 years, IQR 51–70). Median age was 58 years (range 2–92 years, IQR 47–66) for women and 64 years (range 5–89, IQR 56–71) for men. Among all malignancies, breast (185/628; 29.5%) and lung (158/628; 25.2%) cancer were the most common entities. Male patients presented most commonly bone metastases due to lung (98/298; 32.9%) and prostate (50/298; 16.8%) cancer, whereas female patients presented with breast (184/330; 55.8%) and lung (60/330; 18.2%) cancer (Fig. 1). Based on primary cancer grading, 33 (6.0%) patients yielded grade 1, 295 (53.9%) grade 2, and 219 (40.0%) grade 3 tumors.

**Table 1:** Demographic and clinicopathological characteristics of the study groups

Variable	Total (*n*=628; 100%) *n* (%)	PBM^a^ (*n*=93; 14.8%) *n* (%)	PSBM^b^ (*n*=147; 23.4%) *n* (%)	FBM population^c^ (*n*=388; 61.8%) *n* (%)
Age at diagnosis				
Median (range)	61 (2–92)	69 (31–89)	64 (2–92)	58 (5–91)
< 60	299 (47.61)	24 (25.81)	57 (38.78)	218 (56.19)
≥ 60	329 (52.39)	69 (74.19)	90 (61.22)	170 (43.81)
Sex				
w	330 (52.55)	33 (35.48)	73 (49.66)	224 (57.73)
m	298 (47.45)	60 (64.52)	74 (50.34)	164 (42.27)
Tumor grade				
1	33 (6.03)	9 (11.11)	3 (2.33)	21 (6.23)
2	295 (53.93)	42 (51.85)	62 (48.06)	191 (56.68)
3	219 (40.04)	30 (37.04)	64 (49.61)	125 (37.09)
Tumor type				
Breast	185 (29.46)	10 (10.75)	26 (17.69)	149 (36.40)
Lung	158 (25.16)	44 (47.31)	70 (47.62)	44 (11.34)
Prostate	50 (7.96)	13 (13.98)	8 (5.44)	29 (7.47)
Gastrointestinal	65 (10.35)	9 (9.68)	8 (5.44)	48 (12.37)
Genitourinary	32 (5.10)	2 (2.15)	6 (4.08)	24 (6.19)
Head and neck	29 (4.62)	3 (3.23)	8 (5.44)	18 (4.64)
Melanoma	11 (1.75)	1 (1.08)	1 (0.68)	9 (2.32)
Sarcoma	33 (5.25)	4 (4.30)	7 (4.76)	22 (5.67)
Renal	65 (10.35)	7 (7.53)	13 (8.84)	45 (11.6)
Metastasis characteristics				
Osteoblastic	50 (8.06)	5 (5.38)	7 (4.76)	38 (10.00)
Grade 1	5	2	0	3
Grade 2	22	3	2	17
Grade 3	17	0	3	14
Osteolytic	448 (72.26)	67 (72.04)	112 (76.19)	269 (70.79)
Grade 1	15	2	2	11
Grade 2	200	31	42	127
Grade 3	172	26	8	93
Mixed	122 (19.68)	21 (22.58)	28 (19.05)	73 (19.21)
Grade 1	12	5	1	6
Grade 2	72	8	18	46
Grade 3	29	4	53	17
Metastasis localization				
Spine	145 (23.09)	20 (21.51)	38 (25.85)	87 (22.42)
Spine+pelvis+other	227 (36.15)	44 (47.31)	58 (39.46)	125 (32.22)
Spine+other	83 (13.22)	8 (8.60)	21 (14.29)	54 (13.92)
Pelvis	32 (5.10)	1 (1.08)	7 (4.76)	24 (6.19)
Pelvis+other	36 (5.73)	5 (5.38)	7 (4.76)	24 (6.19)
Other	105 (16.72)	15 (16.13)	16 (10.88)	74 (19.07)
Upper extremity	13	2	2	9
Lower extremity	45	8	5	32
Upper extremity+lower extremity	3	-	1	2
Upper extremity+lower extremity+skull	1	-	-	1
Upper extremity+skull	1	-	-	1
Upper extremity+rib	4	1	-	3
Lower extremity+skull	1	-	-	1
Lower extremity+rib	1	-	-	1
Sternum+rib	1	-	-	1
Rib	1	-	1	-
Clavicula	1	-	-	1
Scapula	1	-	-	1
Multiple localizations	32	4	7	21
Complications				
Fractures without neurological complications	315 (50.16)	47 (50.54)	76 (51.70)	192 (49.48)
Fracture with neurological complications	62 (9.87)	9 (9.68)	11 (7.48)	42 (10.82)
Neurological complications without fracture	32 (5.10)	7 (7.53)	7 (4.76)	18 (4.64)
No complications	219 (34.87)	30 (32.26)	53 (36.05)	136 (35.05)
Distribution of fractures^d^				
Fractures	446	62	112	272
Trunk	320	44	89	187
Spine	247	38	64	145
With neurological deficit	31	10	4	17
Paraplegia	11	3	2	6
Without neurological deficit	216	28	60	128
Pelvis	45	3	17	25
Other	28	3	8	17
Extremity	126	18	23	85
Operative treatment of fractures^e^				
Yes	203	39	29	135
No	174	48	27	99

^a^PBM, patients with metastasis diagnosis before primary tumor diagnosis

^b^PSBM, patients with metastasis diagnosis on the same day or within 3 months of primary tumor diagnosis

^c^FBM, Patients with bone metastases at least 3 months after the primary tumor diagnosis

^d^Number of fractures are listed for the different localizations. The number of fractures is not equal to the number of patients as some patients had fractures in multiple localizations

^e^Number of patients in which fractures were treated (or not treated) operatively

Osteolytic lesions were the most common type of BM (72.3%), followed by mixed osteoblastic-osteolytic (19.7%), and osteoblastic lesions (8.1%). Mixed (18/50; 36.0%) and osteoblastic (18/50; 36.0%) BM were seen more frequently in prostate cancer patients. Distribution of BM characteristics in patients with primary cancer grade 1 was 12/32 (37.5%) mixed, 5/32 (15.6%) osteoblastic, and 15/32 (46.9%) osteolytic; in grade 2, the respective values were 72/294 (24.5%), 22/294 (7.5%), and 200/294 (68.0%); and in patients with a grade 3 tumor, distribution was 29/218 (13.3%), 17/218 (7.8%), and 172/218 (78.9%), respectively. In summary, osteolytic BM were more frequently observed in patients with higher, whereas mixed osteoblastic-osteolytic BM were predominant in patients with lower primary cancer grading scores (Table 1, “metastasis characteristics”). The spine was involved in 455/628 (72.5%) of all patients with BM yielding the most commonly affected site.

### Primary bone metastases (PMB) before primary tumor diagnosis

Patients presenting with BM as the first occurrence of diagnosis (93/628, 14.8%) encompassed 35% (33/93) women and 64.5% (60/93) men (Table 1). The most prevalent tumor types in this group were lung (44/93; 47.3%) and prostate cancer (13/93; 14%). 51.9% of PBM patients (42/93) had a primary tumor grade ≤ 2, and 37% of PBM patients (30/93) comprised grade 3 tumors. Osteolytic BM were predominant (67/93; 72.0%), followed by mixed osteoblastic-osteolytic (21/93; 22.6%), and osteoblastic BM lesions (5/93; 5.4%). Localization of BM was distributed equivalent to the total population with the spine as the most commonly affected site (72/93; 77.4%).

### Patients with primary tumor diagnosis on the same day or within 3 months of bone metastasis (PSBM) diagnosis

Of all patients of the study population, 23.4% (147/628) were diagnosed with BM on the same day or within 3 months after primary cancer diagnosis (Table 1). PSBM group comprised 49.7% (73/147) women and 50.3% (74/147) men. Lung cancer (70/147; 47.6%) was the most prevalent cancer type in this group, followed by breast cancer (26/147; 17.7%). 48.1% of patients (65/147) presented with tumor grade ≤ 2, and 49.6% (64/147) had grade 3. Osteolytic BM were predominant (112/147; 76.2%), and the spine was the most prevalent site of BM (117/147; 79.6%).

### Patients with follow-up bone metastases (FBM) at least 3 months after primary tumor diagnosis

The FBM group included 61.8% of all patients (388/628), dividing into 57.7% women (224/388) and 42.3% (164/388) men (Table 1). Among FBM patients, 212 (62.9%) had a tumor grade ≤ 2, and 125 (37.1%) had a tumor grade of 3, respectively. Osteolytic BM lesions were predominant (269/388; 70.8%), followed by mixed osteoblastic-osteolytic (73/388, 19.2%) and osteoblastic BM lesions (38/388; 10%). The spine was the most frequent site of BM and was involved in 266/388 (68.6%) patients in the FBM group (Table 1).

### Complications due to bone metastases - skeletal-related events

**Fig. 2:**
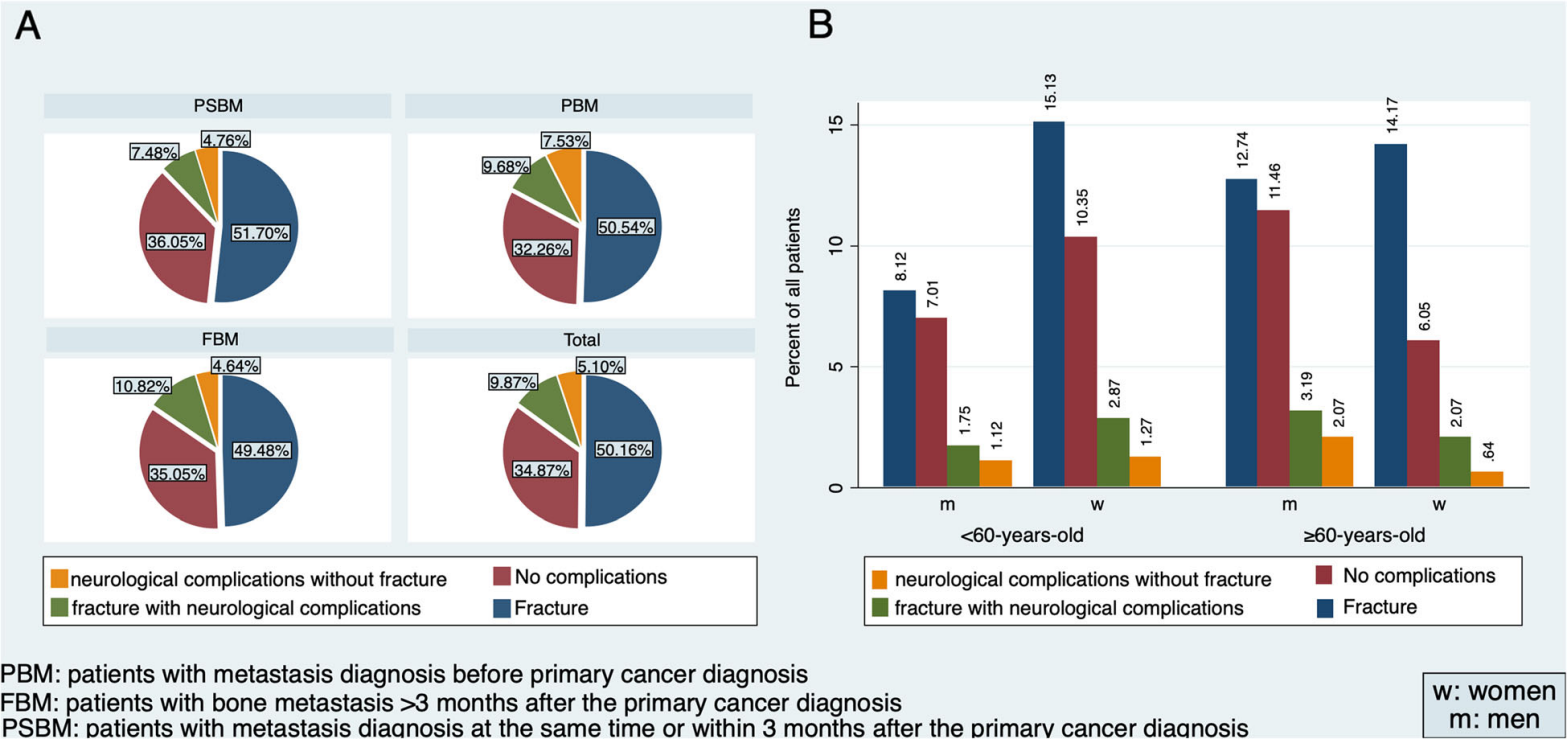
Distribution of complications stratified by study groups (**a**) and sex and age (**b**). The most prevalent complication seen in the study groups were fractures (50.16%) (**a**). Most often, fractures were seen in < 60-year-old women (15.13%), followed by ≥60-years-old women (14.17%) (**b**)

Overall, 409/628 (65.1%) patients had SREs due to BM (Table 1 and Fig. 2). 377/628 (60%) of all patients had fractures, and in 62/628 (9.9%), fractures resulted in neurological deficits. Further, 1.8% (11/628) of patients who had a spinal fracture with resulting neurological deficits suffered paraplegia. Neurological symptoms such as spinal stenosis, which were not a result of fracture, were seen in 32/628 (5.1%) patients. Fractures without neurological complications were more frequently seen in women (184/330, 55.8%) than men (131/298, 44%). Most complications were seen in patients with renal (48/65, 73.9%), genitourinary (23/32, 71.9%), and breast cancer (131/184, 71.2%), whereas lower complication rates were seen in patients with melanoma (4/11, 36.4%), head and neck (16/29, 55.2%), and lung cancer (91/158, 57.6%). Fractures without neurological complications were most often seen in breast cancer (114/184) and genitourinary (19/32, 59.4%) patients. In contrast, melanoma (2/11, 18.2%) and sarcoma (12/33, 36.4%) patients were less frequently affected by fractures without neurological complications. Subgroup analysis with stratification for age (< 60-year-old versus ≥60-year-old) and sex revealed that a similar percentage of patients was affected by SREs in < 60-year-old women (121/186, 65.1%) and < 60-year-old men (69/113, 61.1%), whereas more complications occurred in women (106/144, 73.6%) vs. men (113/185, 61.1%) in the ≥60-year-old population (Fig. 2a). The distribution of SREs dependent on the time point of BM manifestation was similar across the study groups (Fig. 2). However, complications resulting in neurological deficits were slightly more frequent in the PBM group (Table 1). Most frequently, patients underwent nonsurgical therapy (323/628, 51.4%). But notably, a large part of the presented patients underwent combined surgical and local radiotherapy and subsequent chemotherapy (266/628, 42.4%), whereas 39/628 (6.2%) patients underwent surgical therapy solely.

### Metastasis-free survival (MFS)

**Fig. 3:**
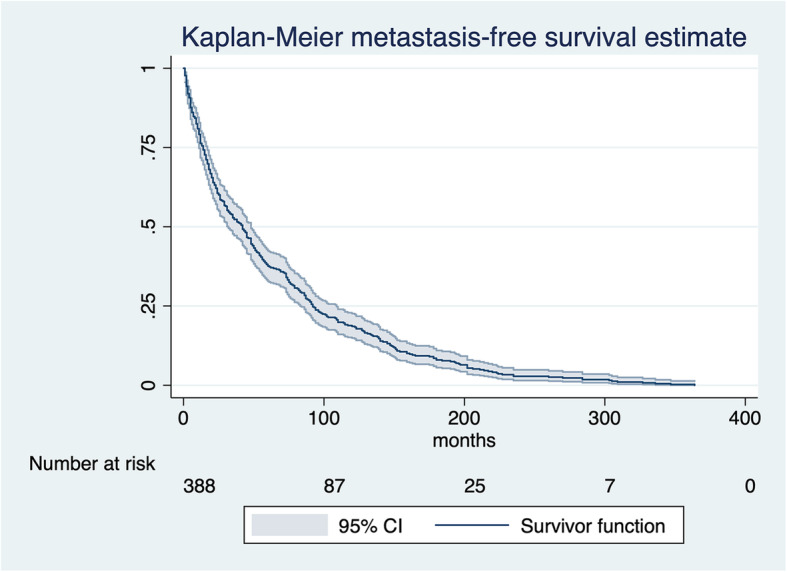
Kaplan-Meier overall survival estimates metastasis-free survival (MFS) probability. The survival probability is plotted against the time to event (primary tumor diagnosis to bone metastasis diagnosis in months). The survivor function is shown with its 95% confidence interval (95% CI). The number at risk at the beginning of the respective x-axis time intervals are shown below the graph. The illustration in the graphs is right truncated for a number at risk ≤5

**Fig. 4:**
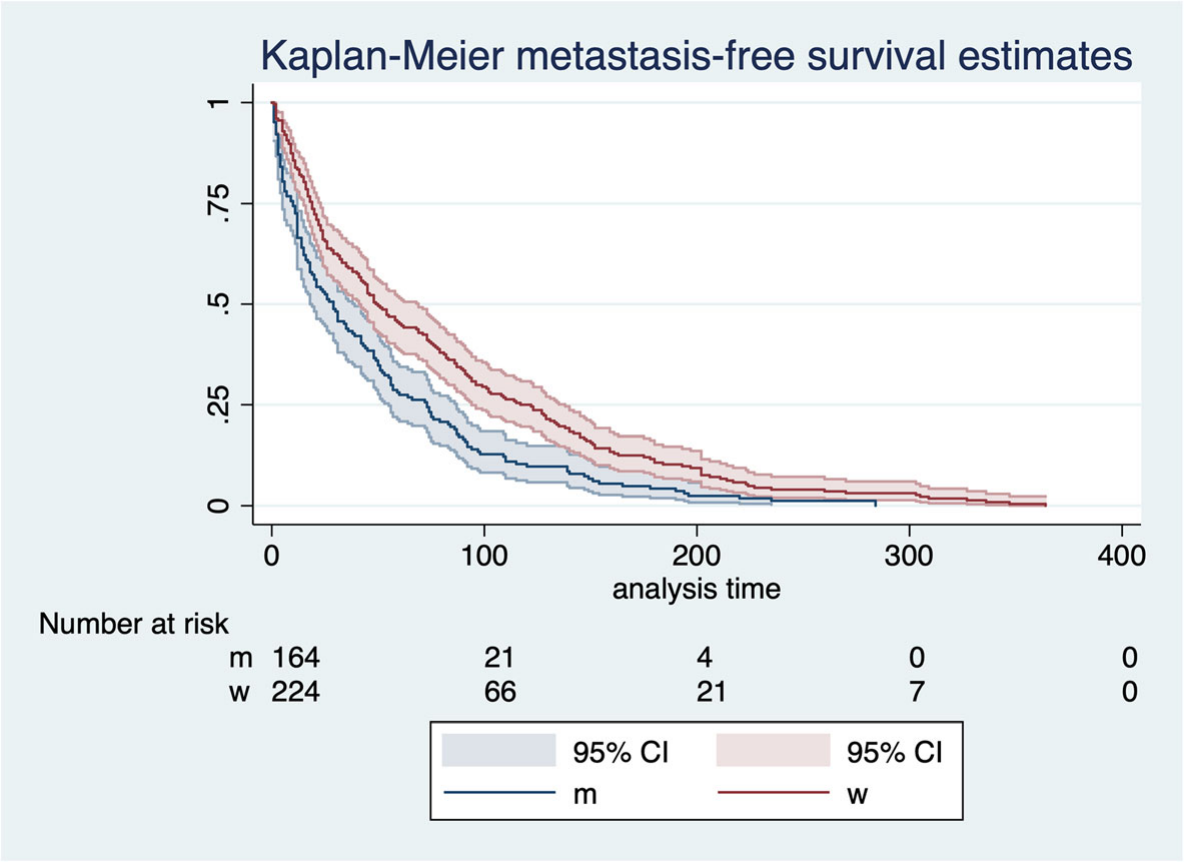
Kaplan-Meier survival estimates metastasis-free survival (MFS) stratified by sex. The survival probability is plotted against the time to event (primary tumor diagnosis to bone metastasis diagnosis in months) for women (w) and men (m), respectively. The survivor function is shown with its 95% confidence interval (95% CI). The number at risk at the beginning of the respective x-axis time intervals are shown below the graph. The illustrations in the graphs are right truncated for a number at risk ≤5

**Fig. 5:**
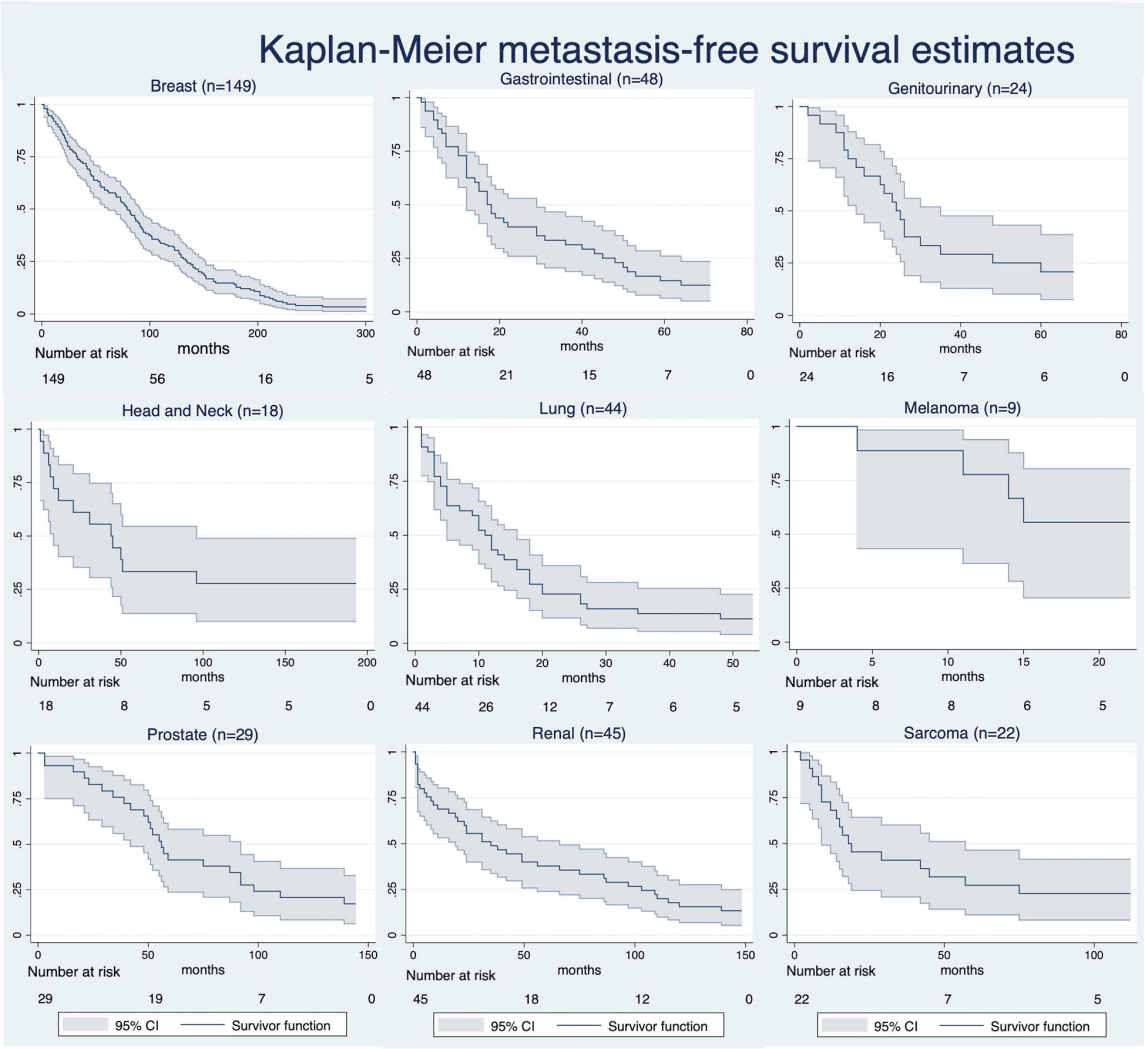
Kaplan-Meier plots for metastasis-free survival (MFS) stratified by tumor type. The survival probability is plotted against the time to event (primary tumor diagnosis to bone metastasis diagnosis in months) for each primary tumor type. The survivor function is shown with its 95% confidence interval (95% CI). The number at risk at the beginning of the respective x-axis time intervals are shown below the graph. The illustrations in the graphs are right truncated for a number at risk ≤5

**Fig. 6:**
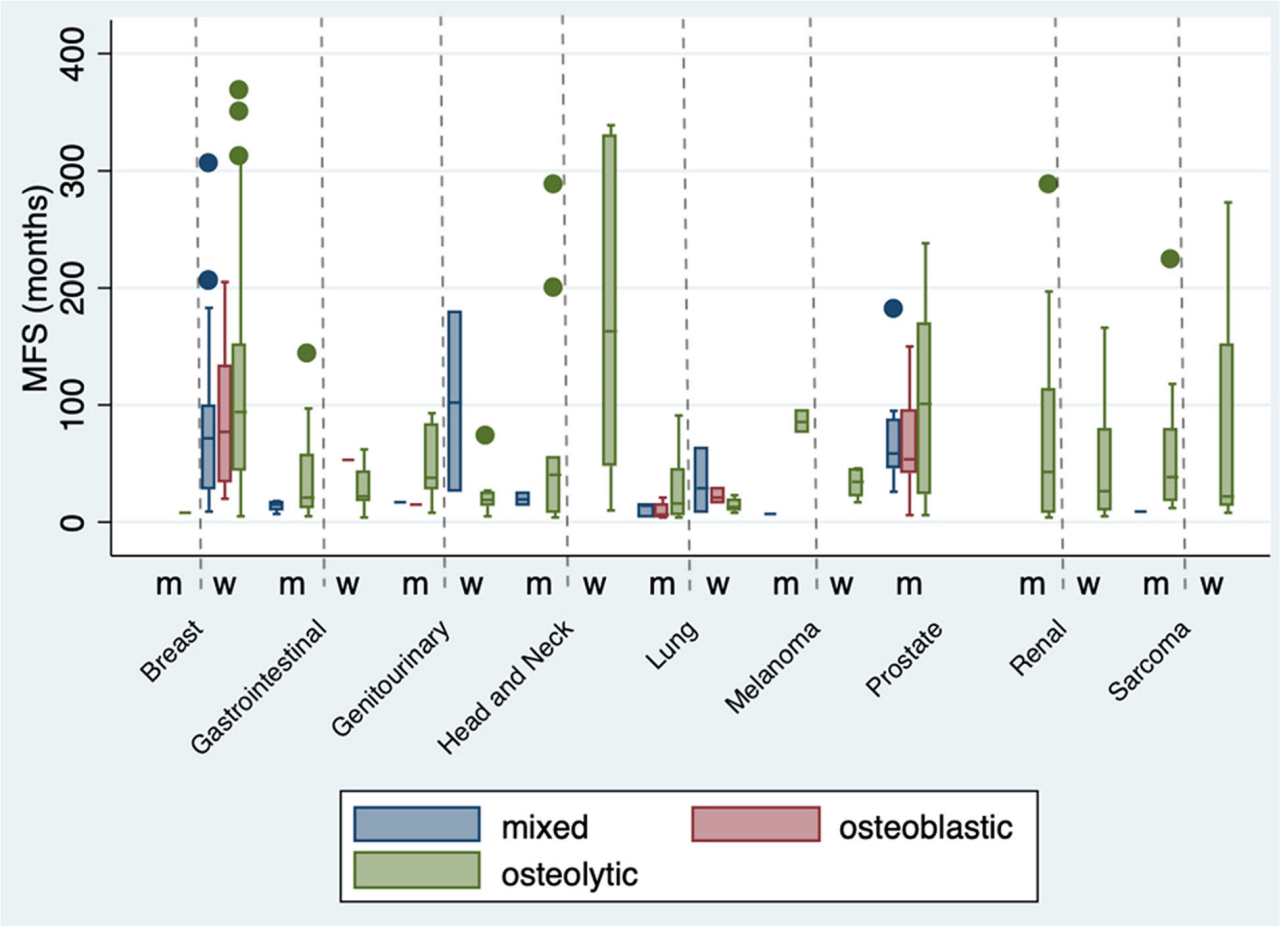
Box plot of metastasis-free survival (MFS) stratified by sex, primary cancer type, and metastases characteristics. The median MFS and its IQR are shown in the box plots grouped by primary cancer type, metastasis characteristics (mixed osteoblastic-osteolytic, osteoblastic, and osteolytic metastases), and sex (women (w) and men (m)). The dots represent outliers

**Fig. 7:**
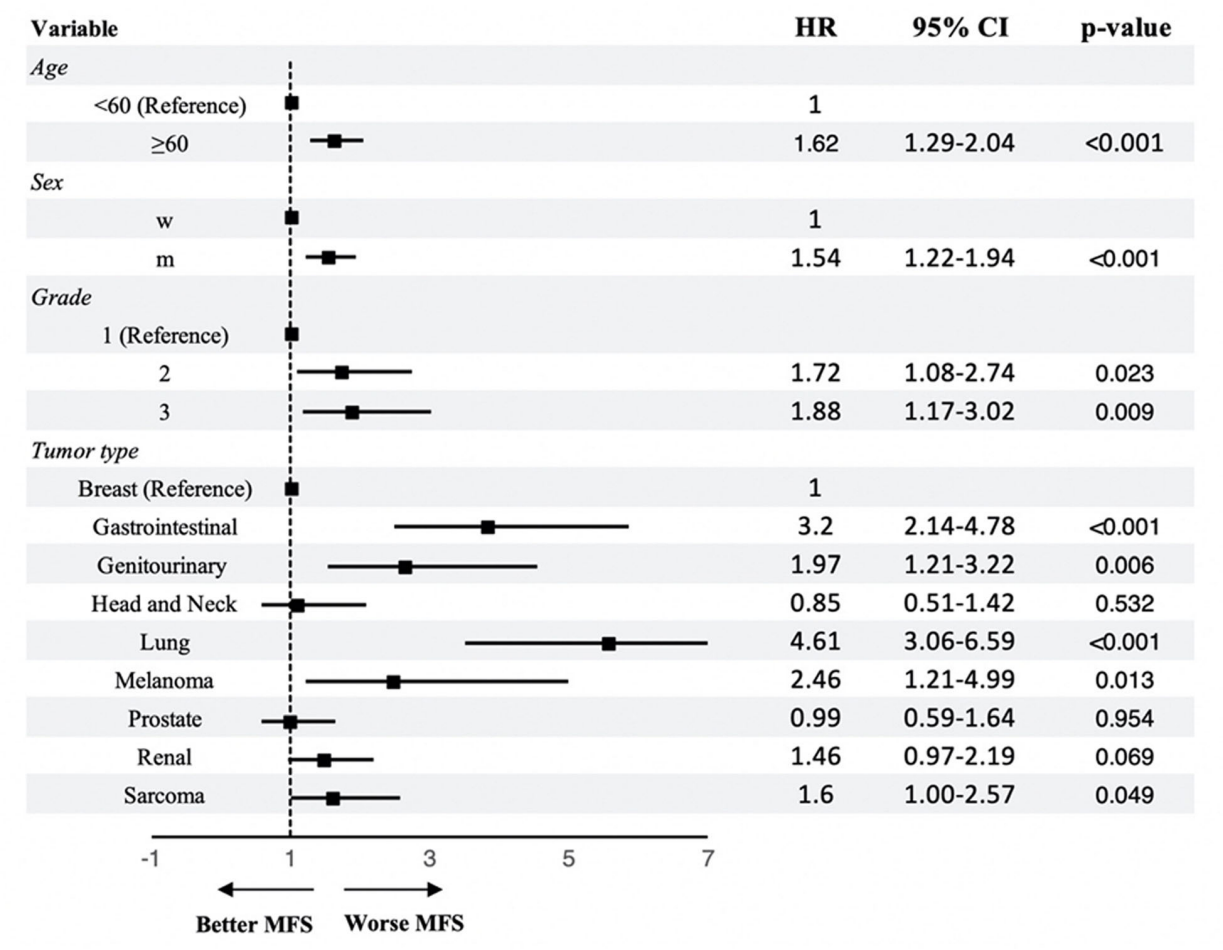
Cox proportional hazard regression model for MFS survival. The hazard ratios (HR) of the respective dependent variables against their references (dummy variables), the 95% confidence interval (95% CI), and the *p*-value adjusted for the included covariates (age, sex, grade, and primary tumor type) in the model are shown

The overall median time interval from primary tumor diagnosis to detection of BM in the FBM group was 45 months (95% CI 35–51) (Fig. 3). Median of MFS was lower in men (32 months, 95% CI 22.0–42.1) vs. women (52.5 months, 95% CI 44.8–72) (Fig. 4). Median of MFS for the different primary cancer types is depicted in Fig. 5 and was significantly different between the primary cancer types: breast (82 months, 95% CI 65.3–94), lung (14.5 months, 95% CI 8.1–19), gastrointestinal (20.5 months, 95% CI 15–3.63), renal (38 months, 95% CI 22.39–74.5), prostate (59 months, 95% CI 49.2–95), sarcoma (21.5 months, 95% CI 14.8–61.3), genitourinary (27.5 months, 95% CI 18.3–42.4), head and neck (47.5 months, 95% CI 12.9–169.8), and melanoma (months 28, 95% CI 14.2–74.6) (*p*< 0.001, log-rank test). Median of MFS was lower for patients with grade 3 (29 months, 95% CI 24.1–44) vs. grade 2 (55 months, 95% CI 42.3–66.1) and grade 1 tumors (86 months, 95% CI 54.3–124.6), respectively. Further, median MFS was lower for ≥ 60-year-old patients (34 months, 95% CI 25.7–44) compared to < 60-year-old patients (56.5, 95% CI 45–73.9) (*p* < 0.001). Figure 6 illustrates median MFS and its IQR via box plots grouped by primary cancer type, metastases characteristics, and sex. Median MFS across was 55 months (IQR 22–90) for mixed osteolytic-osteoblastic, 46.6 months (IQR 21–97) for osteoblastic, and 44 months (IQR 15–97) for osteolytic BM lesions. Median MFS for women suffering from breast cancer was 71.5 months (IQR 29–99) in the mixed osteolytic-osteoblastic BM lesions group, 77 months (IQR 35–132) in the osteoblastic BM group, and 94 months (IQR 45–151) for the osteolytic BM group. Median MFS for men suffering from lung cancer was 14 months (IQR 4–15) for mixed osteolytic-osteoblastic BM lesions, 6.5 months (IQR 5–14) for osteoblastic BM lesions, and 16 months (IQR 6–44.5) for osteolytic BM lesions. The respective values for women were 29 (IQR 8–63), 21 (IQR 16–29), and 13 months (IQR 10–19) for mixed, osteoblastic, and osteolytic BM lesions, respectively. For prostate cancer in men, median MFS was 58.5 (IQR 46–86.5), 53.5 (IQR 42–95), and 101 months (IQR 24–168) for mixed, osteoblastic, and osteolytic BM lesions, respectively. Cox proportional hazard model revealed that an age ≥ 60 years compared to < 60 years (HR 1.48, 95% CI 1.20–1.83, *p*< 0.001) and men compared to females (HR 1.44, 95% CI 1.17–1.78, *p*=0.001) were significantly related to poor MFS. As shown above, a primary cancer grade of 3 compared to a grade 2 or 1 was associated with a poorer MFS when compared in the univariate analysis. This finding was confirmed in our cox regression model after adjusting for age and sex for a grade 3 vs. grade 1 tumor (HR 1.88, 95% CI 1.17–3.02, *p*=0.009) and a grade 2 vs. grade 1 tumor (HR 1.72, 95% CI 1.08–2.74, *p*=0.023) (Fig. 7). Lung cancer (HR 4.61, 95% CI 3.06–6.95, *p*< 0.001), gastrointestinal cancer (HR 3.2, 95% CI 2.14–4.78, *p*< 0.001), melanoma (HR 2.46, 95% CI 1.21–4.99, *p*=0.013), genitourinary cancer (HR 1.97, 95% CI 1.21–3.22, *p*=0.006), and sarcoma (HR 1.60, 95% CI 1.00–2.57, *p*=0.049) were significantly related to lower MFS when compared to breast cancer.

## Discussion

The diagnosis and treatment of bone metastases are challenging due to different time points of manifestation and possible skeletal complications. They have a significant impact on patients’ morbidity and mortality. Hence, thorough knowledge of specific tumor characteristics helps to prevent severe SREs. The present study sought to determine clinicopathologic characteristics, metastasis-free survival (MFS), and SREs of patients who were referred to our orthopedic oncology outpatient clinic at a tertiary center at a comprehensive cancer center. The outcome of the present study reveals that women having breast and lung cancer and men having lung and prostate cancer are at increased risk for MBD and associated SREs. Overall, more than half of the patients had SREs and fractures, respectively, due to BM. Furthermore, our findings suggest that male sex, patients over 60 years of age, and higher primary cancer grading ≥ 2 are predictive factors that are associated with a poorer bone MFS. Most commonly, patients received local radiotherapy and chemotherapy, often combined with adjuvant antiresorptive therapy. Surgery in combination with local radiotherapy and chemotherapy was conducted in nearly half of all patients (266/628, 42.4%), emphasizing the relevance of a multidisciplinary treatment approach.

### Incidence, type, and localization of bone metastases

The reported overall incidence proportion of BM varies among different study cohorts due to the different follow-up times and methodological heterogeneity. It is reported to be 65–75% for breast, 65–75% for prostate, 20–40% for lung, 20–25% for renal, and 2.9–23% for gastrointestinal cancer [15–22]. In a study of 382,733 cancer patients, Hernandez et al. showed that mean time to BM was 400 days (1.1 years), and cumulative incidence of BM was 2.9% (2.9–3.0) at 30 days, 4.8% (4.7–4.8) at 1 year, 5.6% (5.5–5.6) at 2 years, 6.9% (6.8–7.0) at 5 years, and 8.4% (8.3–8.5) at 10 years [23]. The cumulative 1-year, 5-year, and 10-year incidences reported in the literature are shown in Table 2. Harries and Jensen et al. calculated incidence rates instead of cumulative incidences and reported incidence rates of 22 per 1000 person-years, and 172.5 per 1000 person-years, respectively, for BM in breast cancer patients [28, 29]. The incidence rate for breast cancer in our FBM population was 136.96 per 1000 person-years, which is in accordance with the aforementioned. Incidence rates have the advantage of using person-time for each subject and therefore taking into account whenever an event occurs. However, most studies report cumulative incidences that do not account for lost to follow-ups and are therefore less accurate compared to incidence rates, especially for long follow-up time [30]. Nevertheless, our retrospective observational analysis indicated that breast and lung cancer in women, as well as lung and prostate cancer in men, were the most common tumors leading to BM. The distribution patterns of primary tumors among the included patients is in accordance with recently published studies [16, 23, 31, 32].

**Table 2:** Reported cumulative 1-year, 5-year, and 10-year incidence of bone metastases among different tumor entities

Tumor type	1-year incidence (%)	5-year-incidence (%)	10-year-incidence (%)	Reference
Breast	1.9–3.4	3.4–8.4	8.1–12.5	[19, 23–25]
Lung	5.9–10.4	6.3–12.4	12.9	[23, 26, 27]
Prostate	7.7–18	16.6–24.5	29.2	[11, 23]
Gastrointestinal	2.3	3.2	3.6	[23]
Renal	5.8	8.4	9.9	[23]

Osteolytic lesions were predominant in our study cohort, correlating with the results provided by Singh et al. [31]. In a study conducted by Yücel et al., osteoblastic BM lesions were reported to be predominant in patients with lung cancer, prostate, gastrointestinal, and head and neck cancer, whereas osteolytic lesions were predominant in patients with breast and genitourinary cancer [32]. While osteolytic BM lesions are considered to be the most frequent type of bone lesions among different tumor entities, osteoblastic metastases are predominantly found in prostate cancer, which is in accordance with our data [32, 33].

Usually, the spine, ribs, pelvis, and proximal femur are the most common sites for skeletal metastases [34, 35]. In our study, most patients had multiple skeletal lesions rather than solitary ones. As described in other sources, up to 20% of all cancer patients will develop symptomatic spinal metastases [36, 37]. Equivalent to previous reports, the spine was the most frequently involved site of BM in our study [38, 39].

### Bone metastasis-free survival

To avoid under- or overtreatment of patients with BM and help set patient, family, and physician expectations, caregivers need accurate survival estimates. The present study indicated that MFS was different for primary cancer types. MFS was shortest in patients with lung and longest in patients with breast cancer. Numerous recent studies found low bone MFS intervals for lung cancer between 6 and 23 months, which is also in accordance with our data (median 14.5 months, 95% CI 8.1–19). In our study, one breast cancer patient developed BM approximately 31 years after the initial diagnosis of her primary tumor. This extraordinary case highlights the importance of sufficient follow-up visits to cancer patients. About 30% of women with early-stage breast cancer will experience recurrence of disease and develop metastases [40]. Besides regular follow-up visits, new onset of pain must be evaluated, and referral to an orthopedic oncologist should be considered in these cases.

Moreover, sex (male vs. female), age (≥ 60-year-old vs. < 60-year-old), and higher cancer grade were associated with poorer MFS in our study cohort. In a recent study, female sex was proposed to be associated with a lower risk of BM [41]. This prognostic factor was also found to be relevant by Zhang et al., who included these factors in a predictive scoring system [42]. Sex, primary tumor grading, and age were also found to be relevant risk predictors by other recent cohort studies [43, 44].

### Skeletal-related events

Bone metastases are associated with an increased risk of SREs [9, 11, 19, 45]. SREs, including pathological fractures, pain, and spinal cord compression requiring palliative radiotherapy or surgery to bone, occur on average every 3 to 6 months, regardless of the primary tumor entity [15, 46, 47]. Permanent neurological injury and disability can occur via direct tumor compression, bony retropulsion, or deformity due to pathological fracture, or impingement from osteoblastic bone response [6].

Osteolytic BM are more likely to cause fractures than osteoblastic BM lesions, which was also seen in our study cohort [1]. Moreover, 60% (377/628) of our study cohort had pathological fractures, from which 16.3% (62/380) resulted in neurological deficits. Another 5.1% (32/628) of patients suffered from neurological complications of any other cause due to MBD. In a prospective study of 319 patients diagnosed with cancer, the median time interval between back pain and the development of neurological symptoms was 66 days [48]. This study indicates that cancer patients with severe back or spinal nerve root pain need an urgent assessment based on their symptoms.

Predictive risk stratification and structured follow-up programs can, therefore, help to prevent devastating neurological complications resulting from MBD, as an overall of 94/628 (14.97%) patients in our study cohort experienced neurological deficits as a result of metastatic bone disease.

Treatment options for BM and possible consecutive complications include surgery, chemotherapy, and radiotherapy [49]. Further, bisphosphonate and analgesic treatment are integral components of the overall treatment strategy [2]. A large part of the cohort examined in the present study received a combined treatment, including surgery, local radiotherapy, and chemotherapy, respectively (266/628, 42.4%), most commonly combined with adjuvant antiresorptive agents. Orthopedic surgery is not only essential for the treatment of pathological fractures or spinal cord compression. Increasing evidence suggests that prophylactic surgical treatment improves patient outcomes (protective of survival) as pathological fractures of the upper or lower extremity are associated with increased risk for mortality [50]. Hence, profound knowledge on the prognosis and SREs according to the primary cancer type is essential for a successful multidisciplinary therapeutic approach.

## Limitations

The retrospective study design is associated with certain intrinsic limitations, such as selection bias due to the inclusion of a selected cohort (patients at a tertiary orthopedic and trauma center), loss of information, loss of follow-up, variation in imaging techniques, and limited sample size for some tumors. Notably, the present retrospective cohort study focused on complication and metastasis-free survival rates rather than investigating diagnostical or therapeutical approaches. We provided descriptive statistics for nonsurgical and surgical treatment groups in our cohort but did not focus on a more detailed examination of the treatments within these groups. Data regarding surgical management of bone metastases and bone-targeted agents for preventing SREs were sufficiently provided recently by two systematic reviews, involving 59 trials with 10,999 patients [51, 52]. The summarized evidence concluded that patients benefited from the surgical management of bone metastases to the long bones and pelvis/acetabulum, whereas for the pharmacological therapy, denosumab and zoledronate were effective in preventing SREs as well as reducing the risk of pathological fractures and radiation compared with placebo. In contrast, epidemiological data are currently strongly warranted, as there are a limited number of studies focusing on the bone metastasis-free survival rates in primary tumor patients.

## Conclusion

The present study indicates that the incidence and the time point of first bone metastases manifestation vary depending on the primary tumor. Women having breast and lung, as well as men having lung and prostate cancer, are at increased risk for MBD and associated SREs. The time interval of manifestation of BM was lowest in patients with lung cancer and highest in patients with breast cancer. Furthermore, male sex, age ≥ 60 years, and primary cancer grading score ≥ 2 are associated with a poorer bone metastasis-free survival. Intensified follow-up programs for selected cohorts may reduce the risk of SREs and maintain the best affordable quality of life. In conclusion, we call for more extensive prospective cohort studies to close the lack of knowledge regarding bone metastasis-free survival and its underlying influencing factors in cancer patients.

## Data Availability

Datasets are available on request from the corresponding author on reasonable request. The raw data and all related documents supporting the conclusions of this manuscript will be made available by the authors, without undue reservation, to any qualified researcher.

## Abbreviations

ADL: Activities of daily living
BM: Bone metastases
FBM: Bone metastases during follow-up
CI: Confidence interval
HR: Hazard ratio
IQR: Interquartile range
MFS: Metastasis-free survival
MBD: Metastatic bone disease
PBM: Primary bone metastases
PSBM: Primary cancer diagnosis on the same day or within 3 months of bone metastases diagnosis
SRE: Skeletal-related events
UICC: Union International Contre le Cancer
